# Comparison of the Sedative and Cardiovascular Effects of Azaperone and Acepromazine in Dogs

**DOI:** 10.3390/ani16101522

**Published:** 2026-05-15

**Authors:** Marco Antonio de Paz-Campos, Lilia Gutiérrez-Olvera, Héctor Salvador Sumano-López, Graciela Tapia-Pérez, José Antonio Ibancovichi-Camarillo, Uriel López-Parrilla, Williams Alexis Bernal-Palapa, Regina Paola Hernández-Guzmán, Julio Raúl Chávez-Monteagudo

**Affiliations:** 1 Departamento de Ciencias Biológicas, Facultad de Estudios Superiores Cuautitlán, Hospital de Pequeñas Especies, Universidad Nacional Autónoma de México (UNAM), Cuautitlán Izcalli 54740, Mexico; depaz_0@comunidad.unam.mx; 2 Departamento de Fisiología y Farmacología, Facultad de Medicina Veterinaria y Zootecnia, Universidad Nacional Autónoma de México (UNAM), Ciudad de México 04510, Mexico; liliago@unam.mx (L.G.-O.); sumano@unam.mx (H.S.S.-L.); 3 Departamento de Genética y Bioestadística, Universidad Nacional Autónoma de México (UNAM), Ciudad de México 04510, Mexico; tapiadoctora@gmail.com; 4 Facultad de Medicina Veterinaria y Zootecnia, Universidad Autónoma de Sinaloa, San Ángel 3886, Mercado de Abastos, Fraccionamiento San Benito, Culiacán 80260, Mexico; ibanvet@gmail.com; 5 Maestría en Ciencias de la Producción y de la Salud Animal, Facultad de Estudios Superiores Cuautitlán, Hospital de Pequeñas Especies, Universidad Nacional Autónoma de México (UNAM), Cuautitlán Izcalli 54740, Mexico; lopuriel0897@gmail.com (U.L.-P.); williamsalexisbernalpalapa@gmail.com (W.A.B.-P.); 6 Medicina Veterinaria y Zootecnia, Facultad de Estudios Superiores Cuautitlán, Hospital de Pequeñas Especies, Universidad Nacional Autónoma de México (UNAM), Cuautitlán Izcalli 54740, Mexico; guzmanregina316@gmail.com; 7 Departamento de Ciencias Pecuarias, Facultad de Estudios Superiores Cuautitlán, Hospital de Pequeñas Especies, Universidad Nacional Autónoma de México (UNAM), Cuautitlán Izcalli 54740, Mexico

**Keywords:** sedation, dogs, azaperone, acepromazine, cardiovascular

## Abstract

In this study, the degree and characteristics of sedation were assessed using the Grint scale for azaperone and acepromazine in 24 dogs, with the effects on heart rate and systemic blood pressure evaluated simultaneously using oscillometry. Posture, palpebral reflex, eye position, relaxation, response, postural resistance, attitude, systolic pressure, diastolic pressure, mean arterial pressure, and heart rate were analyzed using linear mixed-effects models to evaluate the effects of treatment, time, and their interaction. Significant effects of time were observed for all variables (*p* < 0.001), including systolic, diastolic, and mean arterial pressure, whereas heart rate was not significantly affected by treatment. Based on these results, we conclude that azaperone produces moderate sedation at 20 min after administration, and it does not induce bradycardia or clinically significant hypotension.

## Simple Summary

The sedative effect of azaperone (a butyrophenone) at a dose of 1 mg/kg intramuscularly was studied and compared with that of acepromazine (a phenothiazine). In addition, the heart rate and systemic blood pressure of dogs under the effects of these sedatives were evaluated. It was observed that the sedative effect of azaperone was reached after 20 min without an effect on heart rate or hypotension.

## 1. Introduction

Historically, phenothiazines and butyrophenones have been used in veterinary medicine as tranquilizers/sedatives. In human medicine, they are classified as major tranquilizers due to their use in major psychotic episodes; however, some authors mention that this term is not applicable in veterinary medicine and should not be used [1]. Both phenothiazines and butyrophenones exert their effects on the central nervous system, inhibiting dopamine activity. Dopamine is composed of a catechol radical linked to an ethyl amide and is therefore classified as a catecholamine [2]. Azaperone is a neuroleptic derived from the butyrophenone class, chemically known as 4-fluoro-4-[4-(2-pyridyl)-1-piperazinyl]-butyrophenone [3]. It is commonly used in pigs [4,5], and there are also reports of its use in horses [6]. Although the literature recommends azaperone for use in dogs [7] and some studies have utilized it in canines [3,8], there are no descriptions of its sedation intensity or effects on heart rate and systemic blood pressure. Acepromazine, chemically known as 2-acetyl-10-(3-dimethylaminopropyl) phenothiazine, is commonly used as a sedative and as an anesthetic premedication. Multiple studies have reported on the characteristics and cardiovascular effects of sedation in dogs [9,10,11]. Based on the hypothesis that azaperone produces moderate sedation without significant changes in heart rate or a decrease in blood pressure compared with acepromazine, a prospective, double-blind, randomized clinical trial was established to determine the characteristics and degree of sedation produced by azaperone in dogs. The sedative and cardiovascular effects of azaperone were compared with those of acepromazine. In addition, its effects on heart rate and systemic blood pressure were evaluated.

## 2. Materials and Methods

This study was conducted at the Hospital de Pequeñas Especies de la Facultad de Estudios Superiores Cuautitlán of the Universidad Nacional Autónoma de México, UNAM, and was approved by the Internal Committee for the Care and Use of Experimental Animals (CICUAE-FESC) under registration number C 22_17. Twenty-four healthy dogs were studied. Their health status was determined through medical history, general physical examination, complete blood count, blood chemistry, and urinalysis. All dogs were 1 to 5 years old and weighed 10 to 30 kg. They underwent sedation as part of the pre-surgical management for elective ovariohysterectomy/orchiectomy (as appropriate) and were categorized as ASA I. A random number generator in Microsoft Excel 2024 was used to assign the dogs to the experimental groups. In the group treated with azaperone (AZAP), 1 mg/kg of azaperone (Sural^®^, Chinoin Veterinaria, Aguascalientes, Mexico) was administered, while in the group sedated with acepromazine (ACEP), 0.05 mg/kg of acepromazine (Calmivet^®^, Vetoquinol, Lomas de Chapultepec, Mexico City, Mexico) was administered. The initial doses of azaperone and acepromazine used were the highest reported in the literature. However, azaperone produced excitement in 4 dogs at the initial dose of 2 mg/kg; therefore, it was decided to reduce the dose by half to 1 mg/kg. Both drug preparations were administered intramuscularly into the epaxial muscles without the anesthesiologist being present to assess the degree of sedation and to measure cardiovascular variables. The degree of sedation was initially assessed before the drugs were administered (baseline) and subsequently at 5, 10, 20, 30, 40, and 60 min after drug administration. Heart rate and systolic, diastolic, and mean arterial pressure were recorded simultaneously using oscillometric techniques [12,13,14,15]. The validated Grint sedation scale was used to assess the sedation induced, in which posture, palpebral reflex, eye position, relaxation, response, postural resistance, and attitude were evaluated and assigned numerical values according to the sedation characteristics [16,17]. The researchers who assessed the sedation effects, heart rate, and systemic blood pressure were unaware of the treatment administered to each dog.

### Statistical Analysis

Ordinal (posture, palpebral reflex, eye position, relaxation, response, postural resistance, and attitude) and continuous variables (systolic pressure, diastolic pressure, mean arterial pressure, and heart rate) were analyzed using linear mixed-effects models [18] to evaluate the effects of treatment (TRAT), time (MIN), and their interaction. Given the repeated-measures design, subject was included as a random intercept to account for within-subject correlation. The general model structure was as follows:

Outcome ~ TRAT × MIN + (1 | Subject)

where TRAT represents the treatment group (AZAP or ACEP), and MIN denotes the time in minutes (5.10, 20, 30, 40, and 60). The interaction term TRAT × MIN was included to assess whether temporal changes differed between the two treatments. The model parameters were estimated using maximum likelihood, and statistical significance was set at α = 0.05.

Effect sizes were quantified using both global and local approaches. Marginal *R*^2^ (R^2^m) was used to estimate the proportion of variance explained by fixed effects, while conditional *R*^2^ (R^2^c) included both fixed and random effects. To evaluate the relative contribution of each fixed effect, Cohen’s *f*^2^ was calculated using nested mixed-effects models. Specifically, the effect of treatment was determined by comparing the additive model (TRAT + MIN) with a reduced model including only time; the effect of time was determined by comparing the additive model with a reduced model including only treatment; and the interaction effect was estimated by comparing the full model (TRAT × MIN) with the additive model. Effect size was calculated as follows:

$$f^{2}=\frac{R_{\mathrm{full}}^{2}-R_{\mathrm{reduced}}^{2}}{1-R_{\mathrm{full}}^{2}}$$

Effect sizes were interpreted as <0.02 (very small), 0.02–0.15 (small), 0.15–0.35 (medium), and >0.35 (large).

For ordinal variables, linear mixed-effects models were used as a practical approximation for longitudinal analysis, particularly to evaluate interaction effects. For visualization, ordinal variables were summarized using median and interquartile range (IQR), whereas continuous variables were summarized using central tendency measures across time and treatment groups. Additionally, for continuous variables, post hoc comparisons against baseline (MIN = 0) were performed using a Dunnett-type approach, implemented through mixed models with time as a categorical factor and baseline as the reference level. *p*-values were adjusted for multiple comparisons using the Holm method within each treatment group.

All analyses were performed using Python 3.12.3 (pandas, statsmodels, and scipy libraries) and IBM SPSS 26^®^. Statistical inference was performed using SPSS 26^®^, while effect sizes were calculated using complementary methods, as effect sizes are not directly provided by SPSS for mixed-effects models.

## 3. Results

Significant effects of time were observed across all variables (*p* < 0.001). Treatment effects were significant for several variables, including posture, response, and attitude. Interaction effects indicated that temporal responses differed between the two treatments.

Effect sizes (*f*^2^) ranged from moderate to large, with the largest effects observed for relaxation (*f*^2^ = 1.84) and postural resistance (*f*^2^ = 1.44), indicating strong contributions of fixed effects (Table 1).

Statistical model: Linear mixed-effects models were fitted with treatment (TRAT), time (MIN), and their interaction as fixed effects, and subject as a random effect.

*p*-values: *p*-values < 0.05 were considered statistically significant, with values < 0.001 reported as <0.001.

Heart rate was not significantly affected by treatment (F_1,143.93_ = 0.49, *p* = 0.486), time (F_6,38.85_ = 0.75, *p* = 0.617), or their interaction (F_6,38.85_ = 1.31, *p* = 0.277). Correspondingly, effect sizes were negligible (*f*^2^ < 0.10), indicating minimal contribution of fixed effects.

In contrast, systolic pressure was significantly affected by treatment (F_1,144.21_ = 136.39, *p* < 0.001) and time (F_6,43.27_ = 17.09, *p* < 0.001), while the interaction was not significant (*p* = 0.633). Effect sizes were large for both treatment (*f*^2^ = 1.20) and time (*f*^2^ = 0.85).

Similarly, diastolic pressure showed significant effects of treatment (F_1,138.98_ = 63.95, *p* < 0.001) and time (F_6,37.28_ = 9.14, *p* < 0.001), with no interaction effect (*p* = 0.937). Effect sizes were also large for treatment and time (*f*^2^ = 0.95 and 0.70, respectively).

Mean arterial pressure followed the same pattern, showing significant effects of treatment (F_1,136.53_ = 101.13, *p* < 0.001) and time (F_6,38.10_ = 13.78, *p* < 0.001), and no significant interaction (*p* = 0.850). Effect sizes were large (*f*^2^ = 1.10 for treatment and 0.80 for time).

The mean values ± SEMs for all quantitative variables across time and treatment groups are presented in Table 2.

Heart rate values were comparable at baseline between the two treatments (ACEP: 110.75 ± 3.94 bpm; AZAP: 108.5 ± 5.1 bpm). Over time, heart rate increased initially in both treatments, with a subsequent decrease in the ACEP group and a progressive increase in the AZAP group, reaching 120.58 ± 7.23 bpm at 60 min.

Blood pressure variables showed consistent differences between treatments. The AZAP group maintained higher systolic, diastolic, and mean arterial pressures across all time points. For example, mean arterial pressure at baseline was 111.5 ± 2.83 mmHg (ACEP) versus 133.75 ± 3.75 mmHg (AZAP).

No consistent bradycardic or hypotensive events were observed.

Clear temporal changes were observed in all ordinal variables. In the ACEP group, sedation depth increased progressively, with posture reaching a median of 4.0 (IQR: 3.75–4.0) at 60 min. In contrast, AZAP led to a more moderate and variable response, with posture decreasing to 1.0 (IQR: 0.0–2.0) at 60 min.

Palpebral reflex and relaxation showed similar patterns, with stronger and more sustained effects in the ACEP group compared to the AZAP group.

**Figure 1:**
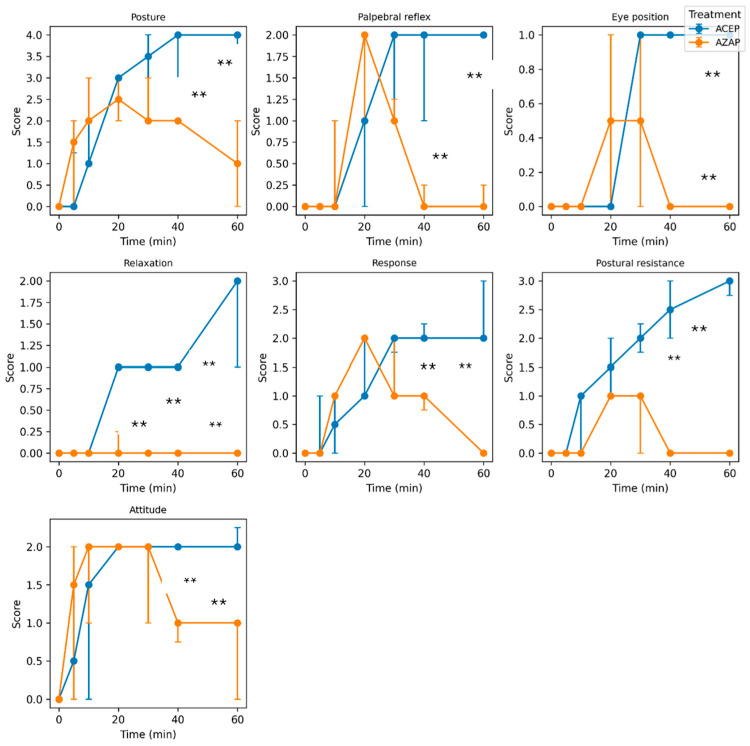
Median values with interquartile ranges (IQRs) of ordinal variables (posture, palpebral reflex, eye position, relaxation, response, postural resistance, and attitude) across time by treatment group (ACEP and AZAP). Error bars represent the 25th and 75th percentiles. Differences between treatments at each time point were evaluated using linear mixed-effects models, with treatment, time, and their interaction as fixed effects and subject as a random effect. Asterisks (**) indicate statistically significant differences between treatment groups (*p* < 0.05).

Figure 1 shows the median values with interquartile ranges (IQRs) for the ordinal variables (posture, palpebral reflex, eye position, relaxation, response, postural resistance, and attitude).

**Figure 2:**
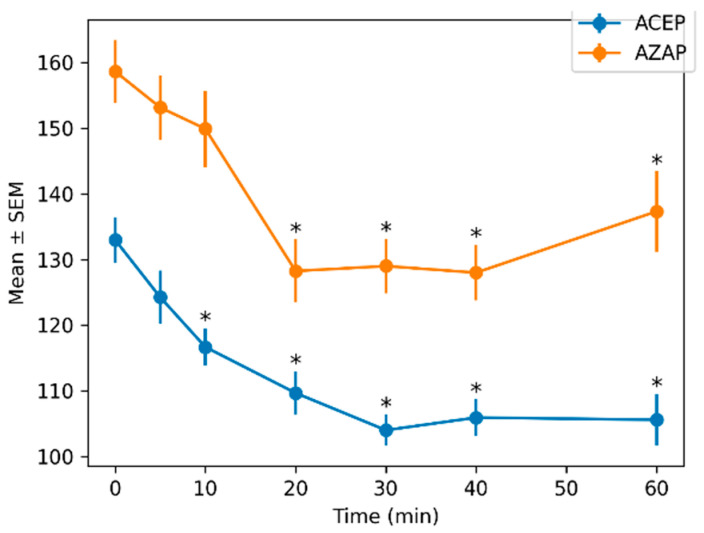
Mean ± SEM of systolic blood pressure across time by treatment. Asterisks (*) indicate significant differences compared with baseline (MIN = 0) within each treatment (*p* < 0.05; Dunnett-type comparisons with Holm adjustment).

**Figure 3:**
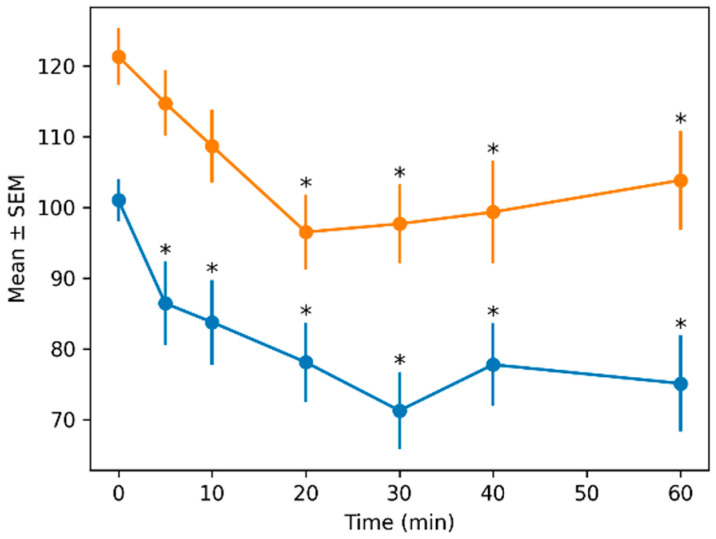
Mean ± SEM of diastolic blood pressure across time by treatment. Asterisks (*) indicate significant differences compared with baseline (MIN = 0) within each treatment (*p* < 0.05; Dunnett-type comparisons with Holm adjustment) Orange (azaperone), blue (acepromazine).

**Figure 4:**
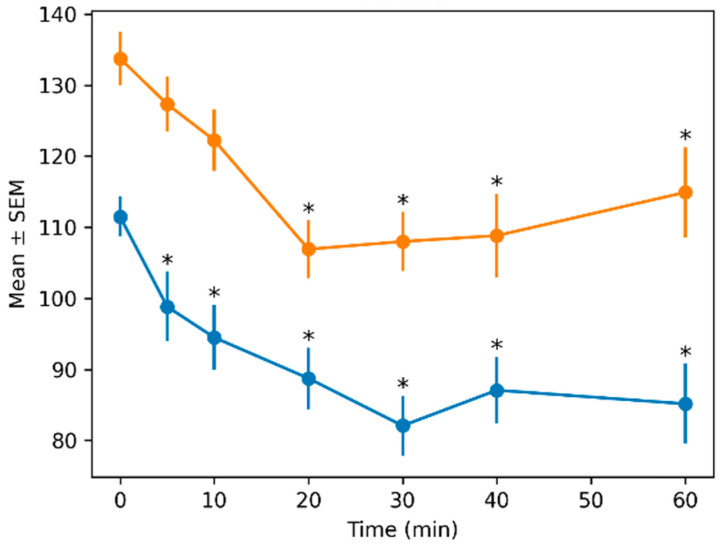
Mean blood pressure, showing mean ± SEM across time by treatment. Asterisks (*) indicate significant differences compared with baseline (MIN = 0) within each treatment (*p* < 0.05; Dunnett-type comparisons with Holm adjustment) Orange (azaperone), blue (acepromazine).

**Figure 5:**
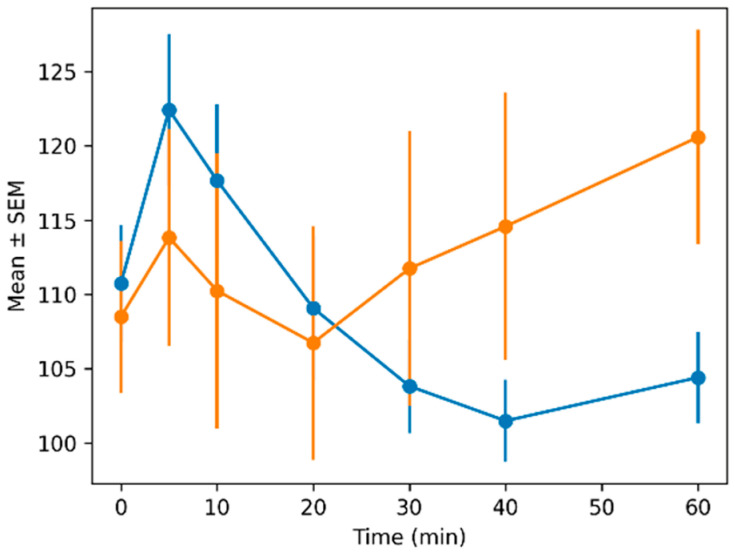
Mean ± SEM of heart rate across time by treatment. Orange (azaperone), blue (acepromazine).

Figure 2, Figure 3, Figure 4 and Figure 5 show the means ± SEMs of quantitative variables across time by treatment.

In this study with dogs, we observed that azaperone, administered at a dose of 1 mg/kg intramuscularly, began to have an effect 5 min after administration and induced moderate sedation at 20 min, but the effect disappeared at 60 min in most of the dogs; thus, the duration of the effect of azaperone at the studied dose was 55 min. In contrast, with acepromazine, the effect was observed at 5 min in six individuals and at 10 min in the other six. The maximum effect occurred at 60 min in 11 dogs and, given the duration of the evaluation in our study, it was not possible to determine the total duration of the effect. However, studies on the sedation time with acepromazine in dogs have determined an effect of up to more than 4 h [19,20]. When evaluating the effect on posture, azaperone caused dogs to lie down within 5 min, whereas acepromazine produced the same effect after 20 min. Comparing the effects of both drugs on the palpebral reflex, it was observed that two dogs in the azaperone group showed an absence of the palpebral reflex after 20 min. Similarly, in the acepromazine group, only two dogs showed an absence of the palpebral reflex, but this occurred at 40 to 60 min after administration. Regarding eye position, dogs treated with azaperone showed no changes in eyeball position, unlike those treated with acepromazine, in which medial strabismus was observed in all individuals. Only one of these dogs presented with strabismus and relaxation of the third eyelid. When tongue and jaw relaxation were assessed, azaperone reduced the jaw tone and induced a moderate swallowing reflex in only three dogs, which occurred at 10 to 20 min after administration. In contrast, acepromazine caused a very reduced jaw tone along with a mild swallowing reflex in two dogs at 40 min, with the same effect observed in another five dogs up to 60 min after administration. In the remaining dogs in this group, acepromazine caused a reduced jaw tone with a moderate swallowing reflex. In the noise response assessment, eight dogs in the ACEP group showed minimal response to the auditory stimulus at 30 min. In the other four dogs, the effect was more intense, and no response to the noise was observed at 40 min. In the AZAP group, minimal response to noise was observed in nine dogs at 20 min. In the remaining dogs, the response was reduced only 10 to 20 min after administration. When resistance to lateral recumbency was assessed, only three dogs in the AZAP group allowed positioning with minimal resistance at 20 min; the rest did not show this effect. In the ACEP group, three dogs allowed positioning without resistance after 30 min and four dogs after 40 min, while the remaining dogs showed no resistance to positioning at 60 min.

## 4. Discussion

Although some reports have described the use of butyrophenones in dogs [21,22,23], this is the first study to report the sedative effects of azaperone in healthy dogs and its effects on heart rate and systemic blood pressure. The effects of azaperone can be compared with those of acepromazine, as the neuroleptic activity of both phenothiazines and butyrophenones is produced by inhibiting dopaminergic activity in subcortical structures [1]. A study comparing the effects of an azaperone/metomidate combination in dogs reported a duration of sedative effect of 50 min, with increased sensitivity to noise, good muscle relaxation, hypothermia, and increased heart and respiratory rates. However, the researchers attributed these findings to the combined effects of both drugs; therefore, it was not possible to identify the specific effects of azaperone in dogs in that study [8]. Azaperone is commonly used in the sedation of pigs, as acepromazine does not produce useful or reliable sedation [24].

In the general appearance assessment, only two dogs in the AZAP group appeared stuporous at 5 min. In the ACEP group, two dogs appeared stuporous at 20 min, and another dog at 60 min. In the AZAP group, only one dog appeared generally normal; the other dogs in this group appeared calm after 10 min. In the ACEP group, nine dogs appeared generally calm at 5 to 10 min. When evaluating the effect of azaperone on heart rate and comparing it with the effect of acepromazine, no statistically significant differences were found. However, both azaperone and acepromazine decreased heart rate from baseline without causing bradycardia. Therefore, it can be concluded that this butyrophenone does not produce a clinically relevant effect on heart rate and has an effect similarly to that observed with acepromazine [25,26,27].

The blood pressure values were higher in the AZAP group than in the ACEP group, with a statistically significant difference. However, despite the significant difference between the two groups, the systolic and mean arterial pressure values obtained for both treatments did not indicate clinical hypotension [28,29]. However, we recommend evaluating the effects of different drugs in conjunction with azaperone, such as inhalational anesthetics, for which there is evidence of hypotension in dogs medicated with acepromazine under isoflurane anesthesia [30,31]. It should be noted that the dose of acepromazine used was determined based on the maximum dose reported in the literature and considering that a dose greater than 0.05 mg/kg does not produce more effective sedation, only prolonging the duration of sedation while increasing the unwanted effects. On the other hand, the dose of azaperone was determined from the only previous study that reported the use of azaperone in dogs, where a dose of 2 mg/kg was administered; however, in our study, we observed intense excitement in three dogs after administration at this dose, and therefore, we determined to decrease the dose. Further studies are needed on the effects of different doses of azaperone in dogs, as it is not yet determined whether its sedative and cardiovascular effects are dose-dependent. One of the limitations of this research is that the effect of the two sedatives on respiratory function was not compared; it will be important to determine if there is a difference between these two drugs. Based on the findings of this research, we must be cautious when considering azaperone as a sedative in humans, since its effects in patients with different pathologies, as well as the quality of recovery, will need to be evaluated. Further studies will be necessary to determine its safety and effect when administered in conjunction with opioids (neuroleptanalgesia) [32,33]. Other limitations of our research include the lack of measurement of plasma concentrations of azaperone and acepromazine at the time of evaluation [34,35]. Furthermore, the only cardiovascular parameters recorded were heart rate and systemic blood pressure; thus, evaluation of more specific cardiovascular parameters will be necessary to determine the full cardiovascular effect. Although a statistical power analysis was performed to determine the sample size, a larger study population will be necessary in future research.

## 5. Conclusions

Azaperone at a dose of 1 mg/kg intramuscularly induced moderate sedation in dogs at 20 min after administration, and did not induce bradycardia or clinically significant hypotension. The duration of the sedative effect was 55 min.

## Figures and Tables

**Table 1:** Abbreviations: N, number of observations; β, regression coefficient; TRAT, treatment effect; MIN, time (minutes); interaction, TRAT × MIN interaction term; *R*^2^ marginal, proportion of variance explained by fixed effects; *R*^2^ conditional, proportion of variance explained by both fixed and random effects; *f*^2^, Cohen’s effect size for fixed effects. Effect sizes (*f*^2^) were calculated using nested models to quantify the relative contribution of treatment, time, and their interaction.

Variable	N	Treatment (β)	*p*-Value	Time (β)	*p*-Value	Interaction (β)	*p*-Value	*R*^2^ Marginal	*R*^2^ Conditional	*f* ^2^
Posture	168	0.77	<0.001	0.06	<0.001	−0.05	>0.001	0.40	0.70	0.68
Palpebral reflex	168	0.54	<0.001	0.04	<0.001	−0.03	>0.001	0.39	0.70	0.64
Eye position	168	0.12	0.08	0.02	<0.001	−0.02	>0.001	0.39	0.70	0.65
Relaxation	168	0.10	0.24	0.03	<0.001	−0.03	>0.001	0.65	0.82	1.84
Response	168	0.34	0.02	0.04	<0.001	−0.03	>0.001	0.35	0.68	0.55
Postural resistance	168	0.01	0.95	0.05	<0.001	−0.04	>0.001	0.59	0.79	1.44
Attitude	168	0.43	0.04	0.03	<0.001	−0.03	>0.001	0.22	0.61	0.27

**Table 2:** Quantitative variables across time by treatment group. Values are presented as mean ± standard error of the mean (SEM). Treatment groups correspond to ACEP and AZAP. Time is expressed in minutes (MIN), with MIN = 0 representing baseline. Differences between treatments and over time were evaluated using linear mixed-effects models, with treatment, time, and their interaction as fixed effects and subject as a random effect. Post hoc comparisons versus baseline were performed using a Dunnett-type approach with Holm adjustment for multiple testing.

Variable	Time (min)	ACEP (Mean ± SEM)	AZAP (Mean ± SEM)
Heart rate	0	110.75 ± 3.94	108.5 ± 5.1
Heart rate	5	122.42 ± 5.12	113.83 ± 7.28
Heart rate	10	117.67 ± 5.13	110.25 ± 9.25
Heart rate	20	109.08 ± 4.87	106.75 ± 7.86
Heart rate	30	103.83 ± 3.15	111.75 ± 9.25
Heart rate	40	101.5 ± 2.77	114.58 ± 9.01
Heart rate	60	104.42 ± 3.06	120.58 ± 7.23
Diastolic pressure	0	101.0 ± 3.04	121.33 ± 4.02
Diastolic pressure	5	86.42 ± 5.91	114.75 ± 4.61
Diastolic pressure	10	83.75 ± 5.95	108.67 ± 5.15
Diastolic pressure	20	78.08 ± 5.58	96.5 ± 5.29
Diastolic pressure	30	71.25 ± 5.46	97.67 ± 5.59
Diastolic pressure	40	77.75 ± 5.84	99.33 ± 7.23
Diastolic pressure	60	75.08 ± 6.78	103.83 ± 7.01
Mean arterial pressure	0	111.5 ± 2.83	133.75 ± 3.75
Mean arterial pressure	5	98.83 ± 4.92	127.33 ± 3.83
Mean arterial pressure	10	94.5 ± 4.61	122.25 ± 4.31
Mean arterial pressure	20	88.75 ± 4.31	106.92 ± 4.04
Mean arterial pressure	30	82.08 ± 4.23	108.0 ± 4.14
Mean arterial pressure	40	87.08 ± 4.69	108.83 ± 5.88
Mean arterial pressure	60	85.17 ± 5.64	114.92 ± 6.36
Systolic pressure	0	133.0 ± 3.47	158.67 ± 4.77
Systolic pressure	5	124.25 ± 4.04	153.17 ± 4.86
Systolic pressure	10	116.67 ± 2.84	149.92 ± 5.79
Systolic pressure	20	109.67 ± 3.32	128.25 ± 4.79
Systolic pressure	30	104.0 ± 2.38	129.0 ± 4.12
Systolic pressure	40	105.92 ± 2.79	128.0 ± 4.26
Systolic pressure	60	105.58 ± 3.91	137.33 ± 6.12

## Data Availability

The data presented in this study are available from the corresponding author upon request.
